# Implementation of Combined Exercise, Fruit Intake, and Vitamin Supplementation Interventions to Prevent Frailty in Older Adults: A RE-AIM QuEST Evaluation

**DOI:** 10.3390/healthcare14193195

**Published:** 2026-09-28

**Authors:** Kun Li, Tingting Shi, Xingyi Huang, Chong Shen, Hui Lu

**Affiliations:** 1 Department of Social Medicine and Implementation Science, School of Public Health, Nanjing Medical University, Nanjing 211166, China; kunli@aliyun.com (K.L.); shidoubleting@163.com (T.S.); 2 Department of Epidemiology, Center for Global Health, School of Public Health, Nanjing Medical University, Nanjing 211166, China; hxy3829@163.com (X.H.); sc100@126.com (C.S.)

**Keywords:** frailty prevention, multicomponent lifestyle intervention, implementation science, RE-AIM QuEST framework, mixed methods evaluation

## Abstract

**Background/Objectives**: Accelerating population ageing has made community-based frailty prevention among older adults an important public health priority. Guided by the Reach, Effectiveness, Adoption, Implementation, and Maintenance Qualitative Evaluation for Systematic Translation (RE-AIM QuEST) framework, this mixed-methods study assessed the implementation outcomes and contextual factors of the multi-component Exercise, Fruit intake and Vitamin Supplementation (EFVF) program and identified factors relevant to its sustainability and potential adaptation to other settings. **Methods**: This implementation evaluation was embedded within a cluster-randomized controlled trial (ClinicalTrials.gov Identifier: NCT06225271) conducted across 14 community clusters in Wuzhong District, Suzhou, China, from March 2024 to April 2025. Quantitative data from participation records, implementation supervision records, and post-intervention follow-up questionnaires were combined with qualitative data from semi-structured interviews with 17 stakeholders, including 3 CDC administrators and 14 frontline primary care providers. Qualitative data were analyzed using a hybrid framework-guided inductive–deductive approach. Quantitative and qualitative findings were analyzed separately and integrated within the RE-AIM QuEST dimensions at the interpretation stage. **Results**: Of the 1380 eligible older adults in the intervention clusters, 475 (34.42%) initiated the EFVF program. Compared with 2248 eligible non-participants, participants showed similar sex distributions (*p* = 0.880) but differed significantly in age (*p* < 0.001), educational level (*p* = 0.002), and marital status (*p* < 0.001). Material incentives, health-seeking needs, community trust, and accessible venues facilitated participation, whereas agricultural work, family responsibilities, limited health literacy, and geographic barriers constrained Reach. Frontline providers perceived improvements in health awareness, dietary practices, exercise behaviors, and social interaction, while also reporting variation in participant responsiveness and limited maintenance of some behaviors after structured support ended. All seven intervention community health centers adopted the program, and 34 of 75 eligible frontline providers participated in delivery (45.33%). All 168 directly observed site-sessions met the prespecified core fidelity criteria, and 393 participants (82.74%) completed both intervention months. At follow-up, provider willingness for continued delivery varied, with 21.43% expressing unconditional willingness to continue the program. **Conclusions**: The EFVF program was implemented with high fidelity under trial-supported community primary care conditions, while frontline workload, behavioral maintenance, and organizational support remained important implementation considerations. These findings support feasibility under the study conditions but do not establish routine-care feasibility or scalability to other settings. Future adaptation should consider local population characteristics, staffing and workflow capacity, flexible delivery arrangements, and sustained organizational support. Multi-site pragmatic evaluation, including implementation cost assessment and longer-term maintenance, is needed before broader implementation can be established.

## 1. Introduction

Global population ageing continues to accelerate, making community-based health management for older adults a key public health priority. According to the 2024 Annual Statistical Communiqué on the Development of China’s Ageing Affairs, China’s population aged 60 years and over has reached 310 million (22.0% of the total population) and 220 million people aged 65 years and older (15.6% of the total population), demonstrating a profoundly deepening trend of population ageing. In this context, frailty, a prevalent and modifiable geriatric syndrome, has drawn growing research and clinical attention. Frailty is characterized by reduced physiological reserves across multiple body systems, resulting in diminished resistance and recovery capacity in response to stressors [1]. Globally, the pooled prevalence of frailty among community-dwelling older adults is 13.3% [2], while national data from China indicate prevalence rates of 10.1% for frailty and 43.9% for prefrailty, with a marked increase in frailty risk with advancing age [3]. Substantial evidence confirms that frailty increases the likelihood of falls, functional decline, and mortality [4,5,6], amplifies the adverse impacts of chronic comorbidities, and imposes heavy burdens on individual families and public healthcare systems [7]. Notably, frailty is not an irreversible outcome of biological ageing but a dynamic and preventable condition. Early identification and targeted intervention, particularly at the prefrailty stage, can effectively delay or even reverse frailty progression and maintain physical function in older adults [8,9].

Current community-based frailty interventions primarily focus on physical exercise, nutritional supplementation, and integrated lifestyle modification strategies [10]. Combined exercise and nutritional interventions have shown synergistic potential for improving physical performance; however, considerable heterogeneity remains in optimal intervention components, dosage regimens, and real-world scalability [11]. Existing nutritional interventions also predominantly emphasize protein and energy supplementation, while evidence supporting multi-dimensional nutritional strategies remains limited [12]. Specifically, few studies have systematically evaluated community intervention models integrating low-to-moderate intensity exercise, regular fruit intake, and vitamin supplementation for frailty prevention. To fill this gap, the cluster-randomized controlled trial (cRCT) titled *Effects of the Combined Intervention of Exercise, Fruit, and Vitamin Supplementation on Frailty in Older Adults* (EFVF) was developed to target community-dwelling older adults with modifiable frailty-related risk factors in eastern China [13].

Despite growing evidence for frailty intervention efficacy, most existing trials prioritize clinical quantitative outcomes while neglecting real-world implementation processes and contextual determinants. Limited in-depth exploration of implementation barriers and facilitators restricts the interpretation of intervention performance and hinders the formulation of optimized, scalable delivery strategies [14,15]. Process evaluation research further indicates that the real-world performance of evidence-based exercise and nutrition interventions is highly dependent on participant adherence, protocol fidelity, staff capacity, resource allocation, organizational support, and local contextual adaptation [16]. Primary care implementation studies also highlight stakeholder engagement, professional competency, service accessibility, and interdisciplinary collaboration as critical determinants of successful frailty intervention delivery [17]. Therefore, systematic evaluation of implementation is essential to clarify how complex lifestyle interventions can be effectively embedded into routine community healthcare practice.

Implementation science increasingly adopts standardized theoretical frameworks to clarify the mechanisms influencing program translation and sustainable delivery in real-world settings [18,19]. The Reach, Effectiveness, Adoption, Implementation, and Maintenance (RE-AIM) framework is widely recognized for its pragmatic capacity to evaluate the public health translation of community programs [20]. The RE-AIM Qualitative Evaluation for Systematic Translation (RE-AIM QuEST) further extends the framework by integrating qualitative inquiry to explain the contextual factors, barriers, facilitators, and mechanisms underlying implementation outcomes [21]. This study reports a comprehensive implementation evaluation of the EFVF program using the RE-AIM QuEST framework. The rationale, setting, and methodological design of the parent cluster-RCT are detailed in the published trial protocol [13], whereas primary quantitative clinical efficacy outcomes are being reported separately in a forthcoming companion publication. Instead, this implementation study focuses primarily on the Reach, Adoption, Implementation, and Maintenance dimensions of the RE-AIM framework and applies qualitative data to assess stakeholder-perceived benefits and the social value of this intervention regarding the Effectiveness dimension.

This study aims to identify key contextual factors shaping EFVF program delivery and to evaluate implementation feasibility under the study conditions while identifying barriers and facilitators relevant to long-term sustainability and potential future scale-up of community-based multi-component frailty prevention. The findings are intended to inform future workflow optimization, standardized tool development, and context-sensitive urban–rural adaptation, thereby providing evidence to guide subsequent evaluation of translation into routine public health practice.

## 2. Materials and Methods

### 2.1. Study Design

We embedded an implementation evaluation within a community-based cluster-randomized controlled trial (cRCT) of the EFVF program (ClinicalTrials.gov Identifier: NCT06225271). Following protocol finalization by expert consensus and a pilot phase (22–24 October 2023), a 12-month trial was delivered across multiple community sites in Suzhou, China (March 2024–April 2025) in collaboration with the Suzhou Center for Disease Control and Prevention (CDC).

Fourteen administrative community clusters served by 13 Community Healthcare Centers (CHCs) were randomized. One CHC covered two distinct community clusters (both allocated to the control arm), while each of the remaining 12 CHCs covered one community cluster. Consequently, 7 intervention clusters were served by 7 distinct CHCs, and 7 control clusters were served by 6 CHCs. Eligible participants were permanent residents aged 65–80 years, independently ambulatory, with frailty-related lifestyle risks (physical activity < 1400 MET-min/week; fruit intake < 50 g/day; no recent B/C vitamin supplementation). Exclusion criteria included fracture history, severe renal/hepatic impairment, neurological disorders, or concurrent trial participation.

Figure 1 provides an overview of the study design and mixed-methods integration process. The implementation evaluation was embedded within the parent cluster-randomized controlled trial and combined quantitative implementation indicators with post-intervention qualitative stakeholder interviews. Quantitative and qualitative data were analyzed separately and subsequently integrated within the five RE-AIM QuEST dimensions to generate dimension-specific interpretations.

### 2.2. Intervention and Setting

#### 2.2.1. Institutional Context and Setting

This evaluation was carried out in the primary healthcare system of Wuzhong District, Suzhou, a developed urban region in eastern China with a mature community public health service network. To ensure convenient participant access and leverage local community trust, the intervention was delivered at 14 randomized community clusters (villages), utilizing existing public service infrastructures including community activity centers and elderly day-care centers. The EFVF program was delivered by frontline healthcare providers from local community health centers (CHCs), serving as the principal program implementers and thereby enhancing the pragmatic relevance of the implementation evaluation. However, delivery remained embedded within a randomized trial with formal supervision and trial-provided resources.

#### 2.2.2. Intervention Components and Delivery

The EFVF program was a multi-component community lifestyle intervention delivered during intervention months 1 and 6 of the trial, with three on-site sessions per week. The intervention comprised four coordinated components: physical activity, fruit supplementation, vitamin supplementation, and health education/communication. Core intervention dose and frequency were standardized across sites, while limited flexibility in exercise modality and fruit variety was permitted within the prespecified protocol. Frontline CHC healthcare providers delivered the intervention in community activity centers or day-care centers under standardized research supervision. Detailed intervention components are summarized in Table 1.

### 2.3. Data Sources and Measures

#### 2.3.1. Data Sources and Collection Procedures

All data were prospectively collected during the formal trial period (March 2024–April 2025) from four independent and complementary sources to ensure multi-dimensional verification of implementation outcomes.

Intervention logs: On-site researchers maintained standardized field logs to record participant recruitment status, baseline demographic information, and session attendance of older adults and CHC staff throughout the intervention.

Supervision logs: Trained research personnel directly observed all 168 scheduled intervention sessions across the seven intervention CHCs (24 sessions per CHC) and completed standardized supervision records. Fidelity assessment focused on compliance with the prespecified core requirements, including intervention frequency, exercise duration, fruit supplementation, vitamin supplementation, and scheduled health education. A session was considered compliant when all required core components were delivered according to the trial protocol. Any protocol deviations and protocol-permitted variations in exercise form, fruit variety, or delivery arrangements were documented in the supervision logs.

Post-intervention questionnaires: At the final 12-month study follow-up, a brief, self-designed 6-item questionnaire developed by the multidisciplinary research team was administered to evaluate the individual-level Maintenance dimension among older adults within the RE-AIM framework. The survey assessed: (1) perceived health benefits of fruit intake and exercise (3-point categorical options: “beneficial”, “somewhat beneficial”, or “not beneficial”); (2) self-reported changes in daily fruit intake and weekly physical activity frequency compared to pre-intervention baseline (5-point Likert-type scales ranging from “Increased markedly” to “decreased markedly”); and (3) adherence barriers for participants without noticeable behavioral increases (structured categorical choices). The questionnaire was administered in Chinese. An English translation containing the complete item wording, response options, and skip instructions is provided in Supplementary File S1. Through convenience sampling, 324 out of the 475 older adults in the intervention arm completed the questionnaire, yielding an overall survey response rate of 68.21%.

Semi-structured key informant interviews: Purposive sampling was used to recruit 17 stakeholders (3 administrators from the Suzhou CDC and 14 frontline providers from the seven intervention CHCs) with direct experience delivering the EFVF program. Eligible participants were required to have participated in intervention guidance or implementation, worked in their current institution for ≥2 years, and provided voluntary consent. Face-to-face semi-structured interviews were conducted by two researchers with a public health background using a guide informed by the five RE-AIM QuEST dimensions and refined through piloting. Interviews lasted 20–40 min, were audio-recorded with consent, anonymized with alphanumeric codes, transcribed verbatim within 24 h, and supplemented with field notes. Sampling continued until information saturation was reached.

#### 2.3.2. Evaluation Indicators and Operational Definitions

All evaluation indicators and operational standards were formulated based on the validated RE-AIM QuEST framework [21,22,23]. Quantitative indicators characterized implementation outcomes, while qualitative interviews explored the contextual mechanisms underlying these findings. Table 2 summarizes the key quantitative indicators and qualitative evaluation focus across the five RE-AIM QuEST dimensions. Detailed operational definitions are provided below.

Reach indicators included the participation rate and demographic representativeness. The participation rate was calculated as the proportion of eligible older adults in the intervention clusters who initiated the EFVF program. Representativeness was assessed by comparing sex, age, educational level, and marital status between EFVF participants (*n* = 475) and eligible non-participants (*n* = 2248).

Adoption was assessed at both institutional and provider levels. Institutional adoption was defined as the proportion of intervention-allocated CHCs that initiated the program, while provider adoption was defined as the proportion of eligible frontline healthcare providers who participated in intervention delivery.

Implementation indicators included protocol fidelity and participant completion. Fidelity was defined as adherence to the trial-specified core dose and schedule, including exercise duration, fruit and vitamin supplementation, and scheduled health education. Protocol-permitted variation in exercise modality and fruit variety was not considered a deviation when the prescribed dose and purpose were maintained. Participant completion was defined as attendance at ≥9 of 12 sessions per intervention month; completion rates for each intervention month, at least one intervention month, and both intervention months were calculated.

Maintenance was assessed at provider and participant levels. Provider-level maintenance was evaluated by frontline providers’ post-trial willingness to continue program delivery, reflecting intention rather than observed routine practice. Participant-level maintenance was assessed using follow-up questionnaire responses on perceived health benefits and sustained exercise and fruit-intake behaviors. “Recognized health benefits” included responses of “beneficial” or “somewhat beneficial,” while behavioral improvement was defined as “Increased markedly” or “Increased slightly” on the corresponding 5-point scales.

### 2.4. Data Analysis

#### 2.4.1. Quantitative Data Analysis

All quantitative statistical analyses were performed using SPSS 25.0 software. Descriptive statistics were used to summarize participant demographic characteristics and program implementation indicators. Normally distributed continuous variables were presented as mean ± standard deviation, and independent samples *t*-tests were used for between-group comparisons. Categorical variables were reported as frequencies and percentages (*n*, %), with between-group differences assessed via Chi-square tests or Fisher’s exact test as appropriate. A two-tailed *p*-value < 0.05 was defined as statistically significant for all analyses.

#### 2.4.2. Qualitative Data Analysis

Qualitative interview data were managed using NVivo version 20.0. A hybrid inductive–deductive content analysis was conducted [24]. Two researchers independently performed open coding to identify emerging barriers, facilitators, and contextual factors, which were grouped into categories and broader themes through iterative discussion. The finalized themes were subsequently mapped to the relevant RE-AIM QuEST dimensions, with cross-dimensional coding permitted where appropriate. Disagreements were resolved by consensus. Transcripts were checked against audio recordings and field notes, and analysis continued until no new themes emerged.

#### 2.4.3. Mixed-Methods Integration

A convergent mixed-methods approach was used within the RE-AIM QuEST framework. Quantitative and qualitative data were analyzed independently and integrated at the interpretation stage within each RE-AIM QuEST dimension. Quantitative findings characterized implementation outcomes, while qualitative findings provided contextual explanations. Integrated findings were summarized in a joint display and interpreted as convergent, complementary, or discordant where applicable.

## 3. Results

### 3.1. Reach

#### 3.1.1. Quantitative Reach Outcomes

A total of 2723 eligible older adults were identified across the 14 randomized community clusters: 1380 in the 7 intervention clusters and 1343 in the 7 control clusters. Within the intervention arm, 475 of 1380 eligible older adults initiated the EFVF program, corresponding to a Reach rate of 34.42%. To assess demographic representativeness, we compared the 475 EFVF participants with 2248 eligible non-participants. The two groups showed similar sex distributions (*χ*^2^ = 0.023, *p* = 0.880), but participants were slightly younger (*t* = 3.553, *p* < 0.001). Significant between-group differences were also observed in educational level (*χ*^2^ = 15.098, *p* = 0.002) and marital status (Fisher’s exact test = 58.376, *p* < 0.001). Participants were more likely to have primary or junior high school education, less likely to be illiterate or semi-literate, more often widowed, and less often married. Detailed baseline comparisons are presented in Table 3.

#### 3.1.2. Qualitative Analysis of Participation Enablers and Barriers

Semi-structured interviews identified five main facilitators of participation: material incentives, health-seeking motivation, social-emotional needs, pre-existing community trust, and geographic accessibility. Free physical examinations and fruit provision encouraged initial participation, while some older adults were motivated by opportunities to obtain health knowledge and preventive guidance. Group-based activities also provided opportunities for social interaction, and established trust in local healthcare providers and convenient community venues further supported participation.

“*Additionally, it includes things like completing a physical examination for free, and then they can also get some food or snacks distributed. This directly brings them in.*”

[JT2]

“*They highly trust us because the relationships between the village residents and our clinical staff are exceptionally good.*”

[GF1]

Participation barriers included time and economic constraints, limited health literacy and institutional distrust, geographic barriers, and family or physical burdens. Seasonal agricultural work and casual income-generating activities reduced available time for participation. Previous public health projects that provided little feedback contributed to skepticism, while dispersed rural settlements and limited transportation created access difficulties. Grandchild-care responsibilities and unexpected injuries further disrupted sustained participation.

“*Our local area has intensive farming seasons where they must do agricultural work. During those peak periods, they simply cannot spare any time… Some of them harvest fruits and have to haul them to local tourist spots for sale.*”

[JT1]

“*If you just tell them to participate in this project, they have no idea what actual benefits it will bring to their bodies in the end, right? Many older adults frequently asked us about past projects: ‘You drew my blood previously, but you never informed us of the final results, so why should we participate in your project this time?’*”

[YX1]

### 3.2. Effectiveness: Stakeholder-Perceived Benefits

Interviewed stakeholders described perceived individual and social benefits of the program, while also noting variation in participants’ responses and difficulties sustaining some behavioral changes.

At the individual level, frontline providers described what they perceived as improvements in health awareness, dietary practices, home-based exercise, and engagement in group activities. Some participants reportedly corrected dietary misconceptions and initiated exercise independently at home, while others gradually shifted from initial reluctance or embarrassment to more active participation. However, perceived responses varied across participant groups. Providers reported less apparent benefit among some rural older adults with high baseline physical activity and among male participants, and noted that behavioral changes tended to weaken after structured supervision ended. The program was also perceived to strengthen frontline providers’ experience in organizing community health activities.

“*Some older adults even told me that they were practicing the exercises independently at home the previous day. They noted that after doing them, their whole body felt lighter and quite comfortable.*”

[XK1]

“*Some residents would tell me: ‘Oh, after listening to what you explained, since I have diabetes, I am now trying my best not to eat heavy or sticky foods for breakfast every day.’ He stopped eating plain rice porridge and replaced it with coarse grains, oatmeal, or millet.*”

[LH1]

At the social level, stakeholders reported that group-based activities provided opportunities for peer interaction, were perceived by interviewees to promote social connection and well-being, and strengthened relationships between community healthcare providers and residents. Repeated face-to-face interaction was also perceived to facilitate trust and routine community health service delivery. Stakeholders further viewed the program as providing practical experience in coordinating healthcare providers, community organizations, and exercise-related resources for community-based frailty prevention.

“*Why are they so eager to visit the day care centers? Because it brings them joy. Staying isolated at home causes distress due to a lack of communication. This collective format makes socialization the priority, which is therapeutic in itself.*”

[LH1]

“*This has profoundly deepened our routine communication and connection with the local populace.*”

[DS1]

### 3.3. Adoption

#### 3.3.1. Quantitative Adoption Outcomes

At the institutional level, all 7 CHCs allocated to the intervention arm formally adopted and initiated the EFVF program, yielding an institutional adoption rate of 100% (7/7). At the individual provider level, 34 of the 75 eligible frontline healthcare providers across these 7 adopting CHCs actively engaged in delivering the health education, exercise supervision, and nutritional guidance sessions, representing a staff-level adoption rate of 45.33% (34/75).

#### 3.3.2. Qualitative Analysis of Adoption Facilitators and Barriers

Stakeholders identified five main facilitators of adoption: administrative support, a simple and operable intervention protocol, stable material and financial resources, community-level collaboration, and positive participant engagement. Leadership endorsement supported organizational participation, while standardized procedures, centralized material provision, and accessible community venues facilitated frontline delivery. Positive participant responses and material incentives were also perceived to reinforce staff willingness to engage.

“*Policy-level endorsement is absolutely essential; administrative attention and leadership support represent a crucial constellation of factors.*”

[JK2]

“*The core strength is operational feasibility. If you are deploying clinical staff or community contract workers to execute a program, it must be straightforward; otherwise, real-world execution fails.*”

[JK2]

Adoption barriers included additional frontline workload, intensive scheduling, unstable implementation settings, fluctuating participant attendance, and concerns about participant safety and institutional liability. Staff described the intervention as an additional responsibility alongside routine public health work, while the intensive schedule increased delivery burden. Venue relocation and seasonal variation in attendance disrupted implementation, and concerns about falls or medical emergencies further reduced willingness to assume intervention responsibilities.

“*A significant portion of this does not fall under their core duties; it represents an entirely extra layer of workload for baseline staff.*”

[YX1]

“*Patient safety is our primary concern. If an older adult experiences a fall or a medical emergency while traveling to or participating in these sessions, the institutional consequences are severe, and none of us can bear that liability.*”

[XK1]

### 3.4. Implementation

#### 3.4.1. Quantitative Implementation Outcomes

Across the seven intervention CHCs, all 168 scheduled site-sessions were directly observed, and all met the prespecified core fidelity criteria, yielding a site-session protocol fidelity of 100% (168/168). At the participant level, 428 participants (90.11%) completed at least one intervention month, and 393 participants (82.74%) completed both intervention months. Site-specific completion rates are presented in Table 4.

#### 3.4.2. Qualitative Analysis of Implementation Facilitators and Barriers

Stakeholders identified three main facilitators of implementation: effective team collaboration, established provider–resident relationships, and immediate on-site incentives. Coordination among community public health staff supported routine program delivery, while existing family doctor networks and familiarity with local residents facilitated participant mobilization and attendance management. Free physical examinations, fruit provision, and group-based activities were also perceived to encourage continued participation.

“*Because my department staff and the community health station personnel maintained excellent cooperation, they were incredibly supportive of whatever task they were asked to do.*”

[GF1]

“*We utilize our family doctor teams. We track who is responsible for which specific zone and how many older adults reside there… Our doctors or nurses are basically well-acquainted with them.*”

[JT1]

Implementation barriers included misconceptions about structured exercise, conflicts between scheduled sessions and participants’ daily responsibilities, and pressures related to on-site crowd management. Some older adults regarded routine housework as equivalent to structured exercise and therefore showed limited motivation for additional training. Household chores, childcare, and personal work also interfered with attendance at fixed session times. In addition, large participant groups and limited staffing occasionally created difficulties in maintaining order during on-site activities.

“*When you tell them how much exercise they need to do each day, they argue, ‘I do the laundry and cook at home, so I’ve already met that.’ They just think their usual routine is active enough.*”

[HJ1]

“*The primary challenge during execution was maintaining field order. Because the number of older adults attending was quite large, it could occasionally become a bit chaotic, and we could only rely on gentle persuasion.*”

[XK2]

### 3.5. Maintenance

#### 3.5.1. Quantitative Maintenance Outcomes

Among the 14 interviewed frontline providers, 21.43% expressed unconditional willingness to continue the program, 50.00% expressed conditional willingness depending on participant adherence or administrative requirements, 14.29% supported continuation only after workload adjustment, and 14.29% were unwilling to participate in long-term delivery.

Among the 324 older adults completing post-intervention follow-up, 80.25% recognized the health benefits of fruit intake, whereas 42.90% reported increased daily fruit consumption; purchasing inconvenience and cost were commonly reported barriers. Similarly, 70.99% recognized the benefits of exercise, while 21.91% reported increased weekly physical activity, with fatigue and insufficient motivation identified as common barriers. These findings indicated greater maintenance of health-related awareness than of sustained behavioral change after structured intervention support ended.

#### 3.5.2. Qualitative Analysis of Conditions for Long-Term Maintenance and Future Adaptation

Stakeholders identified three program features that may support longer-term maintenance and future adaptation: alignment with common health needs of older adults, acceptability of low-impact group exercise, and opportunities for continued health education. The intervention was perceived as relevant to prevalent concerns such as declining physical function and muscle weakness. Traditional low-impact exercise, including Baduanjin, was considered familiar and acceptable to many older adults, while repeated health education was viewed as helpful in reinforcing health knowledge and behavioral awareness.

“*Promotion is highly viable because when we selected this topic, it targeted a primary health crisis currently facing our residents; there is a genuine demand in this area.*”

[JK1]

“*Regarding the activities, we deployed the Baduanjin routine, which carries profound significance for the elderly. Every time, the atmosphere during group practice was excellent.*”

[XK2]

Stakeholders also identified several barriers to long-term maintenance and broader implementation, including urban–rural contextual differences, limited flexibility in intervention arrangements, frontline workload, lack of standardized implementation tools, and insufficient institutional support. Rural settings were perceived as more challenging because of agricultural labor demands, lower health awareness, and dispersed residential patterns. Some stakeholders also noted that intervention content might require adaptation to participants’ physical or dental limitations. In addition, the intensive delivery schedule increased staff burden, while the absence of stable funding, performance incentives, standardized operational tools, and clear cross-departmental coordination constrained sustained institutional delivery.

“*Promotion is much easier in urban neighborhoods because residents live compactly and have lower routine activity levels. Conversely, rural promotion faces distinct hurdles because heavy agricultural labor already demands high physical exertion, and baseline health consciousness is quite low.*”

[JT1]

“*Many rural older adults do not consume fruit due to poor dental health… Future iterations should allow substitutions like cooked vegetables.*”

[HJ2]

“*We need a centralized leading department to draft the overall plan and map out distinct functional lines across sectors.*”

[JK2]

### 3.6. Overall Implementation Summary

The integrated quantitative and qualitative findings across the five RE-AIM QuEST dimensions are summarized in Table 5.

## 4. Discussion

Frailty is associated with loss of independence and adverse health outcomes among older adults [3,25]. However, implementation processes and contextual determinants of community-based frailty interventions remain insufficiently evaluated in primary care settings using standardized frameworks [17]. Using the RE-AIM QuEST framework, this mixed-methods study examined the Reach, Effectiveness, Adoption, Implementation, and Maintenance of the EFVF program, with particular attention to the factors influencing delivery, participation, and sustainability in community practice. The Effectiveness dimension in this implementation report was explored through stakeholder accounts of perceived benefits; quantitative clinical effectiveness outcomes were not analyzed here. The findings highlight both the feasibility of delivering the intervention under trial-supported community conditions and the practical constraints that may affect its adaptation and longer-term implementation in other settings.

### 4.1. Moderate Reach and Contextual Barriers to Participation

The EFVF intervention reached 34.42% of eligible older adults in the intervention clusters. Sex distributions were similar between participants and non-participants, whereas differences were observed in age, education, and marital status. These findings, together with the qualitative data, suggest that participation was shaped by individual availability, health-seeking motivation, family and occupational responsibilities, geographic access, and community trust.

Material incentives, health-seeking needs, familiar community venues, and established relationships with local healthcare providers facilitated participation, whereas agricultural work, family responsibilities, limited health literacy, and geographic dispersion constrained Reach. Choma et al. [26], in a systematic review of physical activity-based fall-prevention interventions for older adults, noted that external-validity indicators such as Reach and representativeness were often incompletely reported, underscoring the importance of examining who participates in community interventions. Boutilier et al. [27] further found that participation among community-dwelling older adults was influenced by personal motivation, communication, and trust in local institutions, which is consistent with our findings on health-seeking motivation and community trust. Wang et al. [28] reported persistent urban–rural disparities in health literacy, supporting our observation that lower health literacy may hinder engagement in rural populations. Karlsson et al. [29] also showed that older adults’ health behaviors are shaped by changing life circumstances and competing daily demands. Together, these findings suggest that extending the EFVF program to other communities will require adaptation to local work patterns, health literacy, accessibility, and social context rather than direct replication.

### 4.2. Stakeholder-Perceived Individual and Social Benefits

Frontline providers described perceived improvements in participants’ health awareness, dietary practices, home-based exercise, and engagement during the intervention, while noting that some changes appeared to weaken after structured support ended. Rookes et al. [30] similarly reported that maintaining behavior change can be difficult for older adults with frailty and multiple long-term conditions. Thapa et al. [31] further found that benefits from a community health worker-delivered lifestyle intervention were not maintained four years after the intervention ended, highlighting the challenge of sustaining gains after structured support is withdrawn. Providers also perceived variation in responsiveness, particularly among older adults engaged in physically demanding agricultural work and among some male participants. Ho et al. [32] reported rural–urban differences in responses to multidomain interventions among older adults, suggesting that baseline lifestyle and activity patterns may influence intervention responsiveness. These findings support considering tailored exercise content and continued low-burden follow-up rather than assuming uniform or sustained responses across populations.

Beyond individual behaviors, providers perceived social benefits from group-based activities, including opportunities for peer interaction and strengthened relationships between residents and local healthcare providers. Such relational and organizational factors are also recognized as important to the implementation of frailty interventions in primary care [17]. For adaptation to other communities, retaining accessible group settings and trusted local provider involvement may help preserve these social and engagement-related functions, although their relevance should be assessed within each local context.

### 4.3. Institutional Adoption and Frontline Capacity Constraints

All seven intervention CHCs adopted the EFVF program, while 45.33% of eligible frontline providers participated in intervention delivery, indicating different adoption patterns at the institutional and provider levels. Stakeholders attributed institutional adoption to administrative support, clear implementation procedures, and available material resources. Previous research has similarly identified leadership and organizational support as important determinants of program adoption and implementation [33].

Frontline providers reported that additional intervention duties competed with existing responsibilities, including chronic disease management, health screening, and administrative work. This finding is consistent with broader evidence that workforce constraints and competing demands can affect primary care implementation [34]. Ginting et al. [15] likewise identified manpower limitations, intervention complexity, and resource demands as barriers to implementing integrated frailty care in Singapore, while organizational support and training facilitated implementation. These findings suggest that adaptation to other settings should consider local staffing capacity, workflow compatibility, and intervention burden before routine integration.

### 4.4. Fidelity to Core Intervention Requirements with Flexible Delivery

All 168 observed site-sessions met the prespecified core intervention requirements, while 90.11% of participants completed at least one intervention month and 82.74% completed both months. The intervention standardized core dose and content while allowing limited flexibility in delivery form, including exercise modality and seasonal fruit selection. This approach is broadly consistent with the Vivifrail model, which preserves core therapeutic components while permitting adaptation to local needs [35].

Stakeholders identified team coordination, established community relationships, and local implementation resources as important facilitators of delivery. Paone et al. [36], in their evaluation of CAPABLE implementation, similarly highlighted the importance of leadership, organizational support, and resources for sustained program delivery. In the EFVF program, familiarity between family doctor teams and local residents also facilitated participant mobilization and attendance management.

Implementation challenges included misconceptions about structured exercise, conflicts with participants’ daily responsibilities, and pressures associated with managing large groups with limited staffing. Ho et al. [32] reported that rural older adults exhibit high, task-oriented activity baselines, requiring interventions to clear cognitive misconceptions to improve compliance. These findings suggest that future implementation in other settings should preserve core intervention requirements while allowing context-sensitive delivery arrangements and adequate staffing support.

### 4.5. Provider and Participant Maintenance Constraints

Maintenance challenges were evident at both provider and participant levels. Among interviewed providers, only a minority expressed unconditional willingness to continue the program, while others reported conditions related to participant adherence, administrative support, or workload. This suggests that continued delivery may depend on how the intervention fits within existing organizational responsibilities and staff capacity. Previous sustainability research has similarly emphasized that long-term implementation is more likely when interventions are aligned with routine workflows and supported by organizational resources [37].

At the participant level, post-intervention tracking showed a gap between perceived benefits and sustained behavior. Although participants acknowledged the intervention’s value, fruit intake and exercise frequency declined once active support ended. This divergence highlights that intrinsic motivation and health knowledge are necessary but insufficient for habit preservation without structural and environmental scaffolds [38,39].

Future implementation should therefore evaluate whether organizational adaptations, long-term monitoring, and integration into routine primary care can support sustained delivery [40]. Broader institutionalization may also require clearer accountability, financing, and resource allocation mechanisms [41].

### 4.6. Considerations for Adaptation and Broader Implementation

This evaluation identified several features that may inform adaptation in other settings, including alignment with older adults’ health needs, use of existing community venues, frontline primary care involvement, and cross-sector coordination. However, the study was conducted in a single district under trial-supported conditions and therefore does not establish scalability. The CDC–CHC–community collaboration should instead be viewed as a context-specific implementation model that may inform adaptation elsewhere.

Broader implementation would require further evaluation of several context-dependent factors. Urban–rural differences may require more flexible schedules and delivery arrangements, while the intensive intervention schedule warrants testing of lower-burden alternatives. Integration with existing primary care workflows may also help reduce additional staff burden. In addition, standardized operational tools may support delivery consistency across sites while allowing appropriate local adaptation of non-core components.

## 5. Limitations

Although this study systematically evaluates the geriatric frailty intervention using the RE-AIM framework, certain limitations remain. First, the trial was exclusively conducted in WuZhong District, Suzhou, with the sample predominantly drawn from rural and peri-urban areas, which may limit the external validity of the findings when extrapolated to regions with different socioeconomic profiles. Future research should expand this geographic scope to encompass diverse primary care populations across varying economic tiers to enhance generalizability.

Second, the study did not include a systematic financial analysis of implementation costs. Incorporating a comprehensive cost-effectiveness evaluation into future research is essential to provide a stronger, data-driven decision-making foundation for policymakers navigating large-scale programmatic scaling.

Third, research personnel directly observed all 168 scheduled site-sessions. Their presence may have encouraged providers to adhere more closely to the intervention protocol. Consequently, the observed 100% site-session fidelity reflects delivery under research supervision; fidelity during routine, unsupervised delivery was not assessed and remains to be established.

Fourth, individual habit maintenance was assessed among 324 of the 475 intervention participants (68.21% response rate) recruited through convenience sampling at follow-up. Because non-completers were not systematically tracked, potential attrition or self-selection bias cannot be fully ruled out if compliers possessed higher intrinsic health motivation. Furthermore, these maintenance outcomes relied on self-reported questionnaires, which are inherently subject to recall and social desirability biases in the absence of objective monitoring devices.

Finally, intentional institutional maintenance was evaluated via interviews with a purposive subset of frontline providers rather than sustained routine practice. Additionally, the qualitative inquiry captured the perspectives of primary care providers and public health administrators rather than older participants themselves; thus, perceived individual-level benefits reflect provider-observed proxy accounts rather than direct experiential narratives from the elderly cohort.

## 6. Conclusions

This RE-AIM QuEST evaluation suggests that the multi-component intervention can be implemented with high fidelity under trial-supported community primary care conditions. Implementation was facilitated by cross-sector coordination, local trust networks, and accessible community delivery, while limited provider participation, frontline workload, and weak maintenance of some participant behaviors remained important challenges.

Future transition toward routine practice would require context-sensitive delivery, streamlined workflows, stronger workforce and financing support, and closer integration with existing primary care services. Multi-site pragmatic studies incorporating implementation costs and longer-term maintenance are needed before broader scalability can be established.

## Figures and Tables

**Figure 1:**
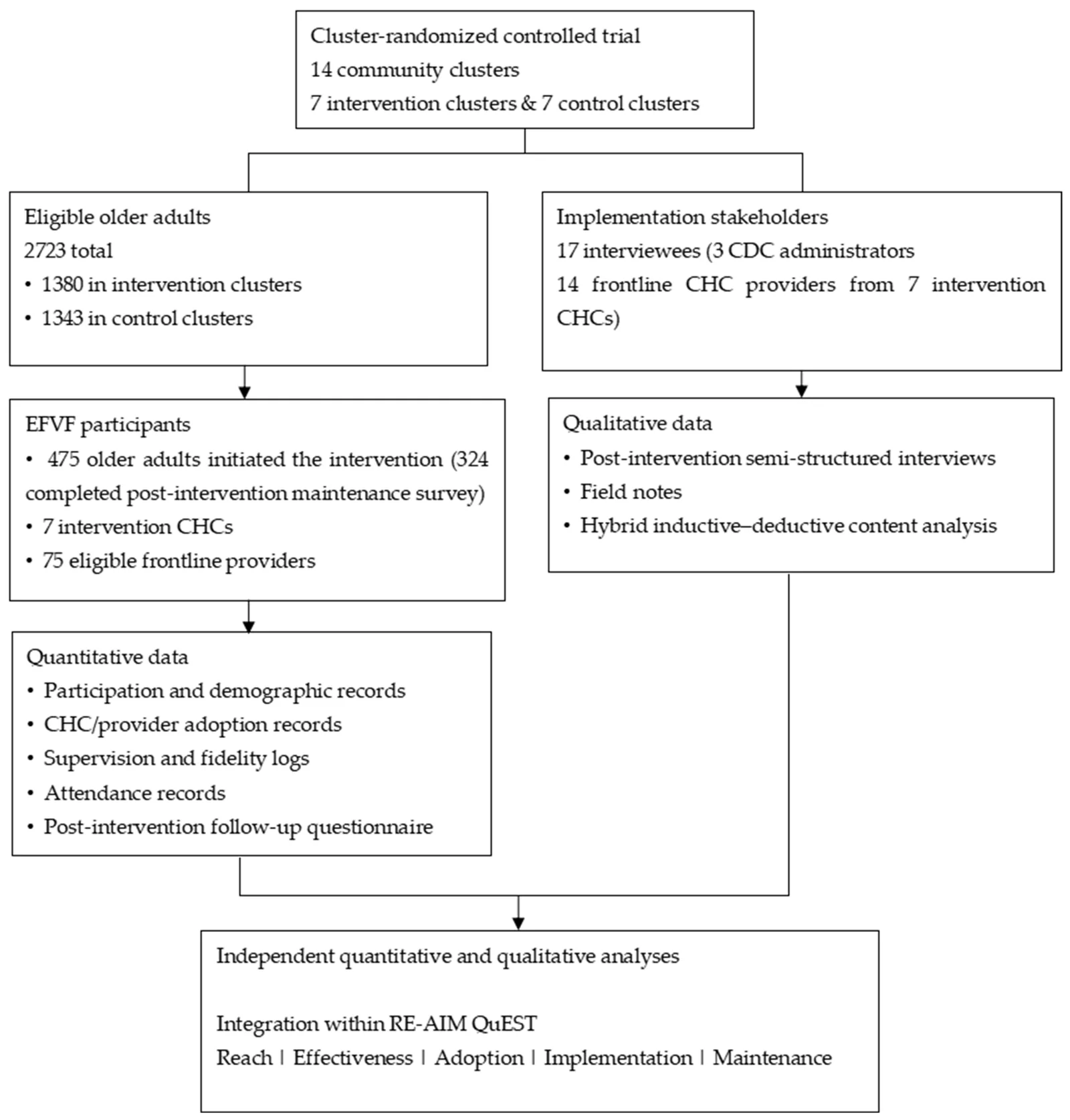
Study design and mixed-methods integration within the RE-AIM QuEST evaluation.

**Table 1:** Structured description of the EFVF intervention components.

Intervention Component	Content	Frequency and Duration	Delivery Personnel	Permitted Protocol Flexibility
Physical activity	Structured low- to moderate-intensity exercise, including aerobic exercise and appropriate alternative exercises for participants with different physical capacities	Three sessions/week during intervention months 1 and 6; ≥30 min/session; 12 sessions/month and 24 sessions in total	Frontline CHC healthcare providers, with research personnel providing supervision	Exercise modality could vary according to physical condition and preference (e.g., aerobic exercise, Baduanjin, grip-ball or seated exercises), while the prescribed duration and therapeutic purpose were maintained
Fruit supplementation	On-site provision and consumption of fresh fruit	≥50 g/session, provided during each on-site intervention session	Frontline CHC healthcare providers	Fruit variety could vary according to seasonal availability and participant preference, while the minimum prescribed portion was maintained
Vitamin supplementation	One vitamin B-complex tablet and one vitamin C tablet daily	Daily during the two intervention months	Frontline CHC healthcare providers provided supplements and monitored adherence	No change to the prescribed vitamin type or dosage
Health communication	Brief frailty-related health communication and encouragement of home-based exercise and fruit intake	Approximately 15 min after each intervention session	Frontline CHC healthcare providers	Local dialect and communication style could be adjusted to facilitate understanding
Structured health education	Group-based education addressing frailty prevention and healthy lifestyle behaviors	At least once per intervention month; ≥45 min/session	Frontline CHC healthcare providers	Presentation format could be adapted locally while core educational objectives were retained

**Table 2:** RE-AIM QuEST evaluation matrix.

RE-AIM Dimension	Quantitative Indicators	Qualitative Focus
Reach	Participation rate; demographic representativeness	Barriers and facilitators to participation; contextual factors influencing Reach
Effectiveness	Not assessed quantitatively in this implementation evaluation	Stakeholder-perceived individual and social benefits; variation in perceived response
Adoption	Institutional adoption rate; provider participation rate	Organizational and workforce factors influencing adoption
Implementation	Protocol fidelity; participant completion	Facilitators and barriers to fidelity and intervention delivery
Maintenance	Provider willingness for continued delivery; participant behavioral maintenance	Conditions for sustained delivery, institutionalization, and future adaptation

**Table 3:** Demographic characteristics of older adults who participated in the EFVF intervention compared with eligible non-participants.

Characteristics	Strata	EFVF Intervention Participants (*n* = 475), *n* (%)	EFVF Non-Participants ^a^ (*n* = 2248), *n* (%)	*t*/*χ*^2^	*p*-Value
Gender	Male	241 (50.74)	1132 (50.36)	0.023	0.880
	Female	234 (49.26)	1116 (49.64)		
Age	Mean ± SD	72.14 ± 3.95	72.87 ± 4.10	3.553 ^b^	<0.001
Educational Level	High school and above	22 (4.63)	84 (3.74)	15.098	0.002
	Junior high school	75 (15.79)	254 (11.3)		
	Primary school	193 (40.63)	837 (37.23)		
	Illiterate or semiliterate	185 (38.95)	1073 (47.73)		
Marital Status	Unmarried	1 (0.21)	3 (0.13)	58.376 ^c^	<0.001
	Married	339 (71.37)	1934 (86.03)		
	Widowed	131 (27.58)	289 (12.86)		
	Divorced	4 (0.84)	22 (0.98)		

^a^ This group included eligible older adults from either intervention or control clusters who did not participate in the EFVF intervention; ^b^ independent samples *t*-test; ^c^ Fisher’s exact test.

**Table 4:** Participant intervention completion rates across 7 community intervention sites (*n* = 475).

Intervention Site ^a^	Enrolled Baseline (*n*)	Intervention Month 1 Completion (*n* ≥ 9 Sessions)	Intervention Month 6 Completion (*n* ≥ 9 Sessions)	At Least One Month Complete, *n* (%)	Both Months Fully Complete, *n* (%)
DS	51	50	48	50 (98.04)	48 (94.12)
GF	56	56	56	56 (100.00)	56 (100.00)
HJ	80	56	51	56 (70.00)	51 (63.75)
JT	84	79	74	81 (96.43)	72 (85.71)
LH	57	57	52	57 (100.00)	52 (91.23)
XK	73	72	66	73 (100.00)	65 (89.04)
YX	74	52	52	55 (74.32)	49 (66.22)

^a^ Site abbreviations are anonymized codes assigned to the seven intervention community health centers.

**Table 5:** Integrated mixed-methods findings across the RE-AIM QuEST dimensions.

RE-AIM Dimension	Main Quantitative Finding	Corresponding Qualitative Finding	Integrated Interpretation
Reach	475/1380 eligible older adults in intervention clusters initiated the program (34.42%). Sex distribution was similar between participants and non-participants, whereas age, education, and marital status differed.	Participation was facilitated by perceived health needs, material incentives, and trust in local healthcare providers, while agricultural work, geographic dispersion, and competing family responsibilities limited participation.	Complementary: Qualitative findings explained contextual factors contributing to incomplete and demographically uneven Reach.
Effectiveness	Quantitative clinical effectiveness outcomes were not analyzed in the present implementation evaluation.	Providers perceived improvements in health awareness, social interaction, and some health behaviors, while also reporting variation in responsiveness across participant groups.	Qualitative-only interpretation: Findings reflect stakeholder-perceived benefits rather than quantitative clinical effectiveness.
Adoption	All 7 intervention CHCs adopted the program (100%); 34/75 eligible frontline providers participated in delivery (45.33%).	Administrative endorsement and material support promoted institutional adoption, whereas additional workload, intensive scheduling, and safety responsibilities constrained staff participation.	Complementary: Strong institutional adoption coexisted with limited provider-level adoption, indicating a gap between organizational commitment and frontline capacity.
Implementation	All 168 directly observed site-sessions met the prespecified core fidelity criteria (100%); 82.74% of participants completed both intervention months.	Team collaboration, established provider–resident relationships, and material support facilitated delivery, whereas intensive scheduling, competing daily responsibilities, and limited staffing created operational challenges.	Complementary: High fidelity and participant completion were achieved despite substantial delivery burden, indicating successful protocol execution under trial-supported conditions rather than absence of implementation barriers.
Maintenance	Follow-up data indicated limited maintenance of some exercise and fruit-intake behaviors; only 21.43% of interviewed providers expressed unconditional willingness to continue the program.	Stakeholders emphasized workload, financing, institutional responsibility, and integration with routine primary care as conditions for sustained implementation.	Convergent: Both data strands indicated that long-term maintenance remained limited and depended on stronger organizational and resource support.

## Data Availability

The interview data are not publicly available due to participant confidentiality and ethical restrictions. Relevant de-identified excerpts supporting the findings are included in the article.

## Supplementary Materials

The following supporting information can be downloaded at: https://www.mdpi.com/article/10.3390/healthcare14193195/s1, File S1: Fruit Intake and Physical Activity Follow-Up Questionnaire.
