# Supplementary material for: Implementation of Combined Exercise, Fruit Intake, and Vitamin Supplementation Interventions to Prevent Frailty in Older Adults: A RE-AIM QuEST Evaluation
Source: Healthcare (Basel). 2026 Sep 28;14(19):3195. doi: 10.3390/healthcare14193195 (PMC13665478; doi:10.3390/healthcare14193195)
Supplement: Supplementary file 1 [file healthcare-14-03195-s001.zip › healthcare-4519487-supplementary.pdf]

## Supplementary File S1: Fruit Intake and Physical Activity Follow-Up Questionnaire

English translation of the six-item questionnaire administered in Chinese at the 12-month follow-up.

| Item | Question and response options |
| --- | --- |
| 1 | **Do you think eating fruit is good for your health?**<br>A. Beneficial<br>B. Somewhat beneficial<br>C. Not beneficial |
| 2 | **How has your average daily fruit intake changed compared with before the intervention?**<br>A. Increased markedly<br>B. Increased slightly<br>C. No change<br>D. Decreased slightly<br>E. Decreased markedly<br>*If A or B, skip Question 3 and proceed to Question 4. If C, D, or E, answer Question 3.* |
| 3 | **If your daily fruit intake has not increased, what is the main reason?**<br>A. No time because of work or farm work<br>B. Fruit is inconvenient to purchase or expensive<br>C. I do not like eating fruit<br>D. Illness or health conditions limit fruit consumption<br>E. Other (please specify): __________ |
| 4 | **Do you think exercising regularly is good for your health?**<br>A. Beneficial<br>B. Somewhat beneficial<br>C. Not beneficial |
| 5 | **How has your weekly physical activity frequency changed compared with before the intervention?**<br>A. Increased markedly<br>B. Increased slightly<br>C. No change<br>D. Decreased slightly<br>E. Decreased markedly<br>*If A or B, skip Question 6 and finish the questionnaire. If C, D, or E, answer Question 6.* |
| 6 | **If your weekly physical activity frequency has not increased, what is the main reason?**<br>A. No time because of work or farm work<br>B. No suitable place to exercise nearby<br>C. Daily activities are already physically tiring / I do not want to be active<br>D. Illness or health conditions limit physical activity<br>E. I do not understand the need for exercise / Other (please specify): __________ |
